# Contributors to the Original Department, Vol. X, of the Buffalo Medical Journal

**Published:** 1855-05

**Authors:** 


*List of Contributors to the Original Department, volume X., of the Buffalo Medical Journal.*

—— Avery, M. D., South Otselic, Chenango Co., N. Y.
Charles H. Baker, M. D., Buffalo.
Prof. Edward H. Barton, M. D., New Orleans, La.
C. Milford Crandall, Belfast, N. Y.
Austin Flint, M. D., Prof. of Theory and Practice in the University of Louisville, Ky.
Frank H. Hamilton, M. D., Prof. of Surgery in University of Buffalo.
John G. House, M. D., Springville, N. Y.
Rev. Charles L. Hequembourg, Dansville, N. Y.
Silas Hubbard, M. D., Buffalo.
William Howell, M. D., Buffalo.
James S. Hawley, M. D., Buffalo.
Sanford B. Hunt, M. D., Prof. of Anatomy in University of Buffalo.
—— Jameson, M. D., South Otselic, Chenango Co., N. Y.
Charles A. Lee, M. D., Prof. of Mat. Med. in University of Buffalo.
Theodore S. McLellan, Esq., Brunswick, Me.
Charles Mayor, M. D., formerly Director of the Hospital of Lausanne, Switzerland.
Edwin R. Maxson, M. D., Geneva, N. Y.
James M. Newman, M. D., Health Physician of the City of Buffalo, 1854.
N. L. North, M. D., Physician to the Williamsburgh Dispensary.
Richard W. Nelson, M. D., Buffalo.
Usher Parsons, M. D., Vice-president Amer. Med. Association.
E. R. Peaslee, M. D., Prof. of Anatomy in New York Medical College and in Bowdin College, Me.
H. A. Potter, M. D., Geneva, N. Y.
W. H. Reynale, M. D., Dansville, N. Y.
John Root, M. D., Health Physician of Buffalo for 1855.
H. M. T. Smith, M. D., Dunkirk, N. Y.; also the
Minutes of Buffalo Medical Association.

To all these gentlemen who have chosen our Journal for the medium of their communications we tender our thanks, and solicit a renewal of their favors.

Among the new features of the Journal during the past year, is the publication of accurate monthly statements of the number and causes of deaths in the city of Buffalo, in immediate juxtaposition with careful and elaborate meteorological statements for the same time. The mortuary record will be continued in its present form, as also the climatic record. It is in contemplation, however, to enlarge the latter on the first of June, and to establish some plan by which its connection with disease may be shown at a glance.

We may be pardoned if at this time we furnish a brief statement of the amount of original, eclectic, and editorial matter in the present volume. It would be an easy matter to carry on a Journal relying on selected matter for its principal supply. But though most of the selected matter offered in the different journals is valuable, it is evident that the best of it may be secured semi-annually in the pages of Ranking or Braithwaite. It should, therefore, assume a secondary place in the pages of a well-conducted monthly, and original communications, by competent writers, (of whom there is no lack, as proved by our list,) and editorial summaries of the current medical news and opinions, should receive the larger space.

There is a certain degree of personality in the description of distinguished medical men, their vagaries, and hobbies, as well as their better qualities, which, conveyed in a pleasant and readable way, though in itself little better than mere gossip, throws an interest about medical subjects as treated by these men, at once attractive and profitable. The stilts of medical dignity grow wearisome after long wearing, and every man has a spice of fun in his composition, which relishes and profits by a free and easy chit-chat about medical men and medical politics—the ambitions of the first, and the constant changes of the latter.

It is in accordance with these views of our editorial mission, and by the help of those contributors of whom we have so much reason to be proud, that we have furnished our subscribers with the following relative amount of material:

| | |
|---|---|
| Original Communications and Reviews, - - - - | 370 pages. |
| Eclectic Department, - - - - - - - - - - - - | 170 " |
| Editorial Department, - - - - - - - - - - - - | 200 " |
| Total reading matter, - - - - - - - | 760 " |

